# Measuring image distortions arising from age‐related macular degeneration: An Iterative Amsler Grid (IAG)

**DOI:** 10.1002/mco2.107

**Published:** 2022-03-04

**Authors:** Inci Ayhan, Edward Doyle, Johannes Zanker

**Affiliations:** ^1^ Department of Psychology Boğaziçi University Istanbul Turkey; ^2^ Department of Ophthalmology Torbay Hospital Torquay UK; ^3^ Department of Psychology Royal Holloway University of London Egham UK

**Keywords:** age‐related macular degeneration, Amsler Grid, iterative method of adjustment, metamorphopsia

## Abstract

Metamorphopsia, perceived as distortion of a shape, is experienced in age‐related macular degeneration (AMD): straight lines appear to be curved and wavy to AMD patients and some other retinal pathologies. Conventional clinical assessment largely relies on asking patients to identify irregularities in Amsler Grids ‐ a standardized set of equally spaced vertical and horizontal lines. Perceived distortions or gaps in the grid are a sign of macular pathology. Here, we developed an iterative Amsler Grid (IAG) procedure to obtain a quantifiable map of visual deformations. Horizontal and vertical line segments representing metamorphopsia are displayed on a computer screen. Line segments appearing distorted are adjusted by participants using the computer mouse to change their orientation in several iteratively such that they appear straight. Control participants are able to reliably correct deformations that simulate metamorphopsia while maintaining fixation in the center. In a pilot experiment, we attempted to obtain deformation maps from a small number of AMD patients. Whereas some patients with extensive scotomas found this procedure challenging, others were comfortable using the IAG and generating deformation maps corresponding to their subjective reports. This procedure may potentially be used to quantify local distortions and map them reliably in patients with early AMD.

## Graphical abstract

We developed an objective method, where using an interactive graphical user interface, participants dragged the intersection point of a cross‐like grid element to the center of the reference square (with different size for different iterations), with the goal to correct deviations from the ideal Amsler Grid. In healthy participants, we verified it to generate quantitative measures of image deformations as experienced in AMD patients. This has the potential to inform future clinical application to assess AMD and guiding interventions, going beyond subjective patient reports.

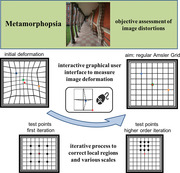

## 1 INTRODUCTION

Age‐related macular degeneration (AMD) is a medical condition, which is characterized by the deterioration of the central region of the retina– known as the macula. In the dry form of AMD, yellow debris, called drusen, accumulate between the retinal pigment epithelium and the choroid layer. In a rarer wet form of AMD, abnormal blood vessels grow beneath the macula, which leak fluid and can also break and leak blood, causing partial loss of vision and metamorphopsia with possible progression and severe damage to the light‐sensitive retinal cells and thus to partial blindness. AMD is the major cause of severe vision loss and the primary cause of blindness for elderly adults of age 70 and over in the western world.^1^ The number of individuals affected by AMD in the UK population is expected to increase progressively from 608,213 to 755,867 within 10 years between 2010 and 2020.^2^ It is estimated that 18% of patients with intermediate AMD and 43% of patients with advanced AMD are at risk of developing late stage choroidal neovascularization (CNV) within 5 years.^3^ CNV often leads to the impairment of sight, accounting for 90% of all cases of severe vision loss caused by AMD. If detected at an early stage, treatment with intravitreal injections of anti‐vascular endothelial growth factor agents improve visual acuity and preserve the visual function of individuals with neovascular AMD.^4^, ^5^ Therefore, early detection of the AMD‐related symptoms is crucial for the preventive intervention.

One of the most commonly used measures of visual function in clinical settings is known to be visual acuity, which is the ability of the eye to discriminate the details of shapes from a given distance. Whereas the human eye is known to be less sensitive to the visibility of visual distortions in natural scenes,^6^ detecting irregularities in repeating patterns, and geometrical shapes seem to be easier. It is mostly the letter charts – rather than natural images ‐ such as Snellen acuity or more recent early treatment diabetic retinopathy study (ETDRS) charts which are used to measure the degree of disease progression in patients suffering from the vision loss. AMD patients have been reported to perform poorly in letter identification tasks, which indicates a reduced spatial resolution capacity of their visual system.^7^ As well as a reduction in visual acuity, though, they also suffer from another condition called metamorphopsia, which manifests itself as the perceived distortion of the shape and slant of objects. In the presence of metamorphopsia, straight lines appear to be curved and wavy to the sight of a patient. As a consequence of such image distortions, one of the most significant impairments for AMD patients is the progressive loss of reading ability, which can be only partially managed with reading aids that facilitate eccentric reading.^8^ The main cause of metamorphopsia has been suggested to be the displacement of the photoreceptors in the sensory retina due to retinal contraction of proliferating membranes and the detachment of the retinal pigment epithelium from Bruch's membrane of the choroid^9^. A better understanding of this disease, particularly in relation to the pace and severity of the deterioration, is critical in managing this condition.

Wiecek et al.^10^ have studied the effect of metamorphopsia on acuity by measuring the letter identification performance as a function of spatial scale and distortion magnitude, as well as the letter size. Interestingly, they found that the letter identification was influenced by the interaction between the spatial scale of the distortions and the letter size ‐ such that distortions at high spatial frequencies were particularly disruptive for the letter identification of larger rather than smaller letters, and performance deteriorated for smaller letter sizes with greater distortions, which together highlights the importance of measuring spatial distortions while evaluating the visual acuity of patients who also experience distortions in shape. It is known that the low‐spatial frequency processing is fairly preserved in patients with AMD and that the deficit is specifically found in the band of higher spatial frequencies.^11^, ^12^ Tested using natural images, in a recent study by Peyrin et al.,^13^ it has also been demonstrated that irrespective of the luminance contrast levels, the error rates of AMD patients in a scene categorization task are higher for scenes with higher spatial frequencies rather than lower special frequencies, whereas various manipulations on natural image contrast are known to play a rather insignificant role on the visual search behavior of patients with central vision loss.^14^ All of these findings provide evidence that the spatial scale at which changes in perceptual distortions emerge due to structural changes in the retina might have differential influences on changes in acuity; thus, having a reliable and quantifiable map of metamorphopsia, as well as the performance in acuity tasks, is crucial in evaluating the progression of the eye diseases.

Computer‐based algorithms may help improve the sight of visually impaired people while performing daily tasks such as reading. Correction methods of this sort, which are based on computer‐mediated reality, enhance vision by making use of the digital image processing techniques which compensate for perceptual decline. Jacko et al.,^15^ for example, used a feature‐enhancing software to demonstrate that whereas a simple magnification does not provide a useful strategy to improve the visual search performance in patients with low vision, background color, the number of graphical elements on an interface, and the size of the graphical image all contribute to the success of the image enhancement methods. The literature on the image enhancement methods provides rather mixed results, however, and although some improvement has been reported in the reading speed of the visually impaired patients using such selective digital image processing methods,^16^ most have failed to show any benefit on either reading speed,^17^ or face recognition,^18^ and visual search accuracy.^14^ For the implementation of more successful computerized correction methods, there seems to be a need to personalize the corrections by obtaining first a reliable, accurate map of individual deformations and then applying the image enhancement methods according to the individual needs.

Currently, the most common clinical tool to assess metamorphopsia is the Amsler Grid, which is composed of equally spaced straight lines lying along the vertical and horizontal axes. Typically, a 10 cm × 10 cm grid comprising 0.5 cm squares is held at a reading distance with one eye covered. At a reading distance of 45 cm, these dimensions correspond to the size of the central 12.5 degrees of the visual field. Therefore, any distortions or missing areas on the grid are taken as a sign of a problem with the macula. Despite its common use, however, the Amsler Grid does not provide a quantitative measure of the severity of distortions and can usually be used as a solely qualitative diagnostic tool (i.e., presence or absence of metamorphopsia). In a recent study, though, Wiecek, Lashkari, Dakin, and Bex (2015) provided an estimation of metamorphopsia in a large group of AMD patients which had been tested on the Amsler Grid test by taking the ratio of distorted pixels to the total number of pixels in the digitalized images. Their analyses demonstrated that wet AMD patients are approximately 3.65 times more likely to experience metamorphopsia than dry AMD patients. They also showed that the distortions are more likely to exist in the central than in the peripheral vision of wet AMD patients.

To measure the degree of metamorphopsia in epiretinal membrane (ERM) patients, Matsumoto et al.^20^ have developed a method called M‐Charts, which is based on the observation that lines with coarser dot intervals are perceived to be less deformed than the lines with finer dot intervals. Having presented a series of lines with different dot intervals, the minimum visual angle of the dotted lines needed to cause the metamorphopsia to disappear was taken as a measurement to indicate the severity of the condition. M‐charts are not the only tools that take advantage of the relationship between the densities of dot arrays and the detection thresholds of visual distortions. Using a similar logic, in the method called D‐Charts, patients are asked to report irregularities on an octagonal array of small black squares arranged at different eccentricities, while keeping their fixation on a central red dot.^21^ Once the reports are collected on one particular configuration, the regions of distortions are then converted into lower‐density arrays, where detecting the irregularities gets harder, a procedure which is repeated until the patient stops seeing the distortions. Initial attempts of practitioners^21^ to apply two‐dimensional grids of dots in octagonal frames (D Charts) to identify regions of distortion have been successful in demonstrating the effects of macular hole surgery in some patients but still suffer from limited quantitative detail and precision, which would be essential for the design of tools to improve reading ability in AMD patients using computer‐based algorithms such as augmented‐reality systems.

Yet another method to investigate macular lesions is the preferential hyperacuity perimetry (PHP) which is a psychophysical test based on hyperacuity (vernier acuity) judgments.^22^ In this procedure, pathological distortions are quantified on the basis of patients’ responses to artificial distortions of varying sizes. Follow‐up studies, however, have shown that although the sensitivity of the PHP to detect CNV in recently diagnosed patients is high (82%),^23^ the rate of false positives is also significant such that 18% of patients without AMD are detected to have AMD‐related lesions in the PHP test.^24^ All these tests require eye fixation in the center of the visual field, yet they did not track the eye positions. Although such assessments intend to monitor disease progression, it has proven difficult to localize distortion and quantify distortion across participants.

Martin‐Gonzalez et al.^25^ have recently introduced a method which allows the user to report local deformations on an interactively deformable grid. This method relies upon the verbal description of the deformations by the examinee, which is then corrected by the examiner using an interpolation method on the digital image. In order to analyze the reliability of the system, the researchers first simulated metamorphopsia in healthy participants by tracking their gaze and presenting a particular pattern of deformation on the very same region of the visual field during the task. Participants were then instructed to report the location and orientation of the abnormalities verbally so that the examiner, blind to the distortion of the participant, would be able to deform the distortion in the opposite direction of the perceived curves using digital image processing tools until the simulated distortion would appear as straight to the eye of the participant. In a similar computerized task, Wiecek et al.^26^ introduced a method, where the description by the participants with regard to the extent and shape of distortion on the grid was corrected by the experimenter using a computer mouse. Spatial context, however, is known to change form perception. In both of these methods, participants were always presented with complete grids with deformations on particular regions. How the performance is affected by different scales of deformations and in the absence of the overall context (i.e., grids on the non‐deformed regions) is a question yet to be answered.

Here, we introduce, and test in two versions, a computer‐based method called “iterative Amsler Grid” (IAG) in which the deformations in different regions of the visual field are tested locally and separately in iterations of a simple adjustment of probe dots in a similar grid. The method is based on patient‐computer interaction using a mouse and a cursor. Separate regions of the overall grid are tested at different spatial scales in the absence of peripheral reference grids. Our current purpose is to test the feasibility and reliability of the procedure in participants with normal vision. If the deformation fields can be precisely mapped, image correction methods can be applied in the future to compensate for individual distortions in AMD patients, in an attempt to restore visual function.

## 2 RESULTS

### 2.1 Experiment 1: IAG ‐ *Line alignment paradigm*

Standard Amsler Grids, which are used to diagnose metamorphopsia in clinical settings, cannot provide a topographic map of visual deformations. Thus, the aim of experiment 1 is to demonstrate the feasibility of a new computer‐based method to measure the pattern and overall magnitude of perceived image deformations ("metamorphopsia") in a control group without visual impairment making use of a simulated metamorphopsia condition.

**FIGURE 1:**
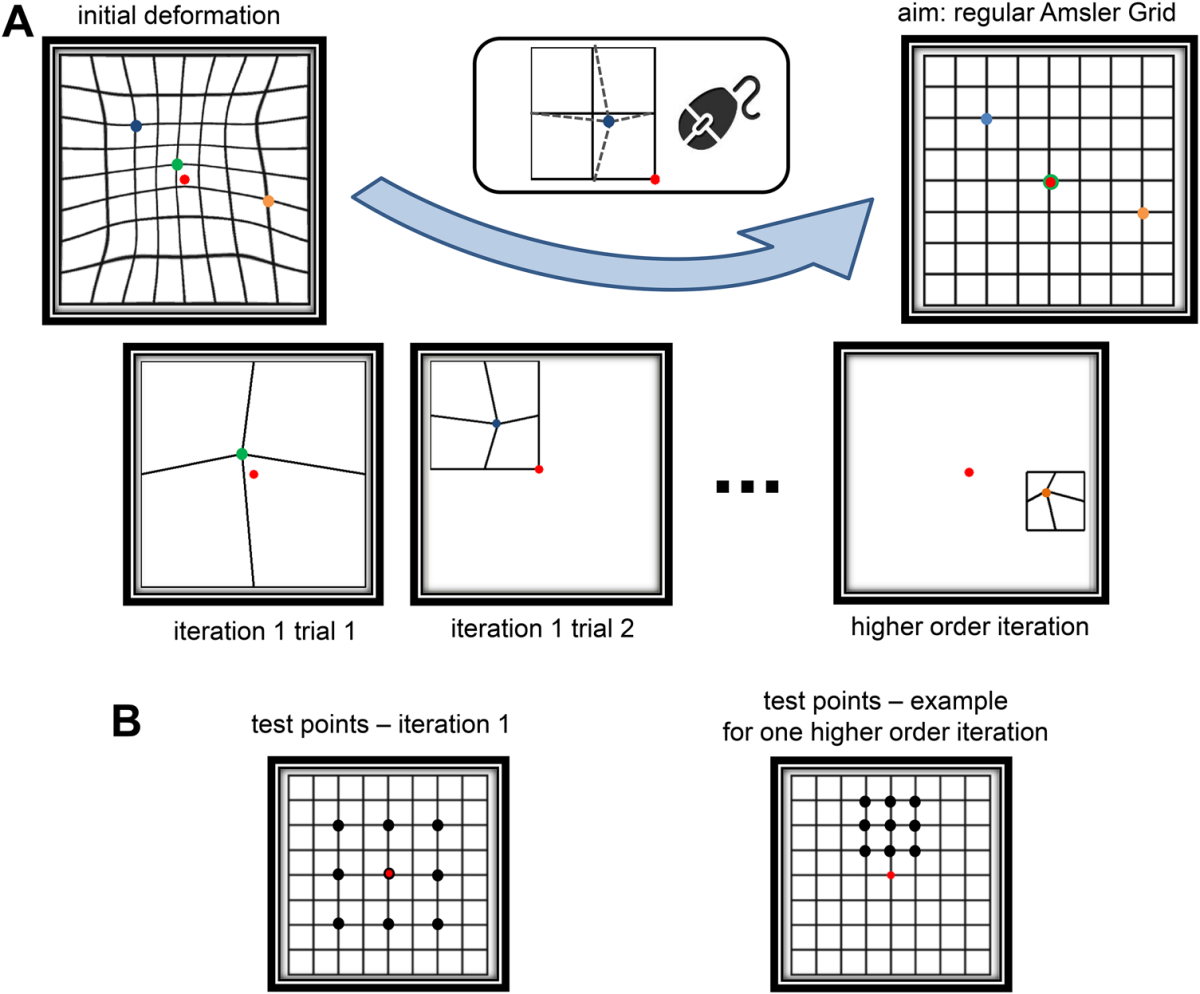
(A) Visual depiction of experiment 1, where using an interactive graphical user interface, participants were dragging the intersection point of a cross‐like grid element (e.g., green, blue, and orange dots and dashed lines in (A)) to the center of the reference square (with different size for different iterations), with the goal to correct deviations from the ideal Amsler Grid. Red dot indicates the central fixation point. (B) Location of the test points at different eccentricities and scales (iterations). The aim of participants was to achieve a complete regular grid in the course of iterations. Each iteration consisted of nine trials, with test points sitting on the adjacent regions of a 3 × 3 grid

IAG is based on a procedure to probe local image deformations, which form part of a global Amsler Grid, in iterations that optimise the sampling of test regions across the full stimulus region – the final set of local corrections can be used to present an Amsler Grid that appears straight and regular to the individual observer and to generate an individual map of perceived local deformation and an overall deformation score (Figure 1; Movie S1)

**FIGURE 2:**
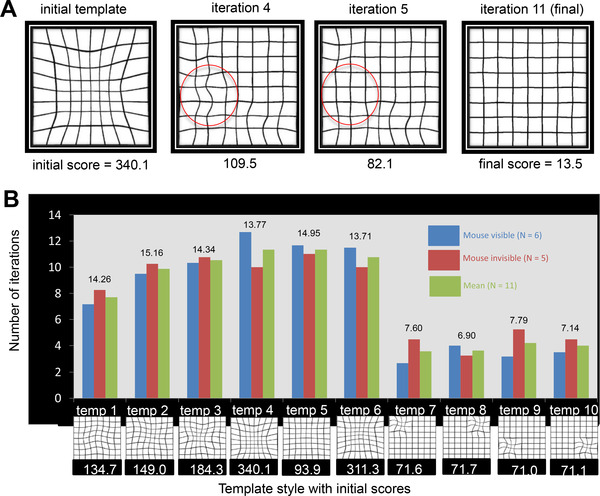
(A) Example of interpolated Amsler Grids across iterations, which participants used to select the next regions for further and finer corrections. Note how the deformed grid elements inside the red circle in iteration 4 were corrected in iteration 5. Numbers below the grids indicate the error measures in each iteration. (B) The main results of the line alignment paradigm of experiment 1 for mouse‐cursor‐visible (white bars) and mouse‐cursor‐invisible (gray bars) conditions, together with the overall means of the two conditions (black). Whereas in the mouse visible condition, the mouse cursor was visible on the screen; in the mouse invisible condition, it was absent from sight, the comparison of which could give us an insight about the role of attention. The average number of iterations required to correct for irregularities is plotted as a function of different template styles with widespread (templates 1–6) and local (templates 7–10) deformations. Numbers above the bars indicate the mean (*N* = 11) y‐axis values

To have a quantitative measure of the deformations, we calculated an error measure value which computed the sum of squares of the distances between the shifted and the veridical points for 81 node points of an 8 × 8 grid. Figure 2A demonstrates an example performance through iterations in which the error measure decreases gradually as the participant makes further and finer corrections. As it can be seen, the grid on the final iteration is a good approximation to a non‐deformed grid. In Figure 2B, we plot the number of iterations and the final error measures averaged across all observers as a function of the template style and the initial error values. A paired *t*‐test, where we compared the average number of iterations it took to complete single‐region templates (templates 7–10) to the number of iterations it took to complete template 5, showed that although they have similar initial error measure values, correcting deformations on a single region requires significantly less many iterations (*M*_MouseVisible _= 3.33_,_ SD_MouseVisible _= 1.78; *M*_MouseInvisible _= 4.30_,_ SD_MouseInvisible _= 2.41) than correcting a widespread deformation on template 5 (*M*_MouseVisible _= 11.67_,_ SD_MouseVisible _= 2.42; *M*_MouseInvisible _= 11.20_,_ SD_MouseInvisible _= 1.64) in both mouse visible, *t*(5) = −5.85, *p* = 0.002, Cohen's *d* = −2.39, and mouse invisible conditions, *t*(4) = −8.08, *p* = 0.001, Cohen's *d* = −3.61. This finding implies that it is not the severity but rather the distribution of the deformations, which determines the number of iterations for participants to complete the task. In fact, the conditions in which only a single region of a grid was deformed (Temp 7, Temp 8, Temp 9, Temp 10) took significantly less iterations to complete (*M*_MouseVisible _= 3.33_,_ SD_MouseVisible _= 1.78; *M*_MouseInvisible _= 4.30_,_ SD_MouseInvisible _= 2.41) than the grids with widespread deformations of the image (Temp 1, Temp 2, Temp 3, Temp 4, Temp 5, Temp 6) (*M*_MouseVisible _= 10.47_,_ SD_MouseVisible _= 1.21; *M*_MouseInvisible _= 10.30_,_ SD_MouseInvisible _= 0.79) in both mouse visible, *t*(5) = ‐10.6, *p* < 0.001, Cohen's *d* = −4.31, and mouse invisible conditions, *t*(4) = ‐5.19, *p* = 0.007, Cohen's *d* = −2.32.

A 2 (mouse visible and mouse invisible) × 2 (templates 1–6: widespread deformations and templates 7–10: local deformations) Mixed ANOVA, with mouse visibility as the between‐ and template style as the within‐subjects factor demonstrated that mouse visibility did not make a significant change in the number of iterations to correct for the simulated deformations, *F*(1,8) = 0.33, *p* = 0.582, ηp^2^ = 0.040, which may suggest that the presence of the mouse cursor does not draw attention from the resources allocated for the correction task. Confirming our paired‐samples *t*‐test results between the regional and more scattered deformations, Mixed ANOVA analysis also showed a significant effect for the main template styles, *F*(1,8) = 92.619, *p* < 0.001, ηp^2^ = 0.920, such that regional deformation templates were found to be corrected in less iterations than the grids with deformations widespread the image. The interaction between the mouse visibility and the template style, on the other hand, was not found to be significant, *F*(1,8) = 0.312, *p* = 0.592, ηp^2^ = 0.038.

#### 2.1.1 Gaze stability

In a control experiment, we measured the eye movement of two participants (two of the authors) running through the IAG procedure, using a Tobii X120 infrared eye tracker (sampling gaze positions at a rate of 60 Hz), in order to verify the stability of their gaze position on the fixation point at the center of the grid. The ages of the participants were 29 and 55 years old. For calibration, an undistorted Amsler Grid was used, where participants had to fixate steadily on each of the four corners and the center. Gaze data were then collected for all nine test fields in five regions of iteration 2, and for each gaze point, the distance of gaze position to the position of the fixation target and to the test point that was adjusted by the participant was calculated.

**FIGURE 3:**
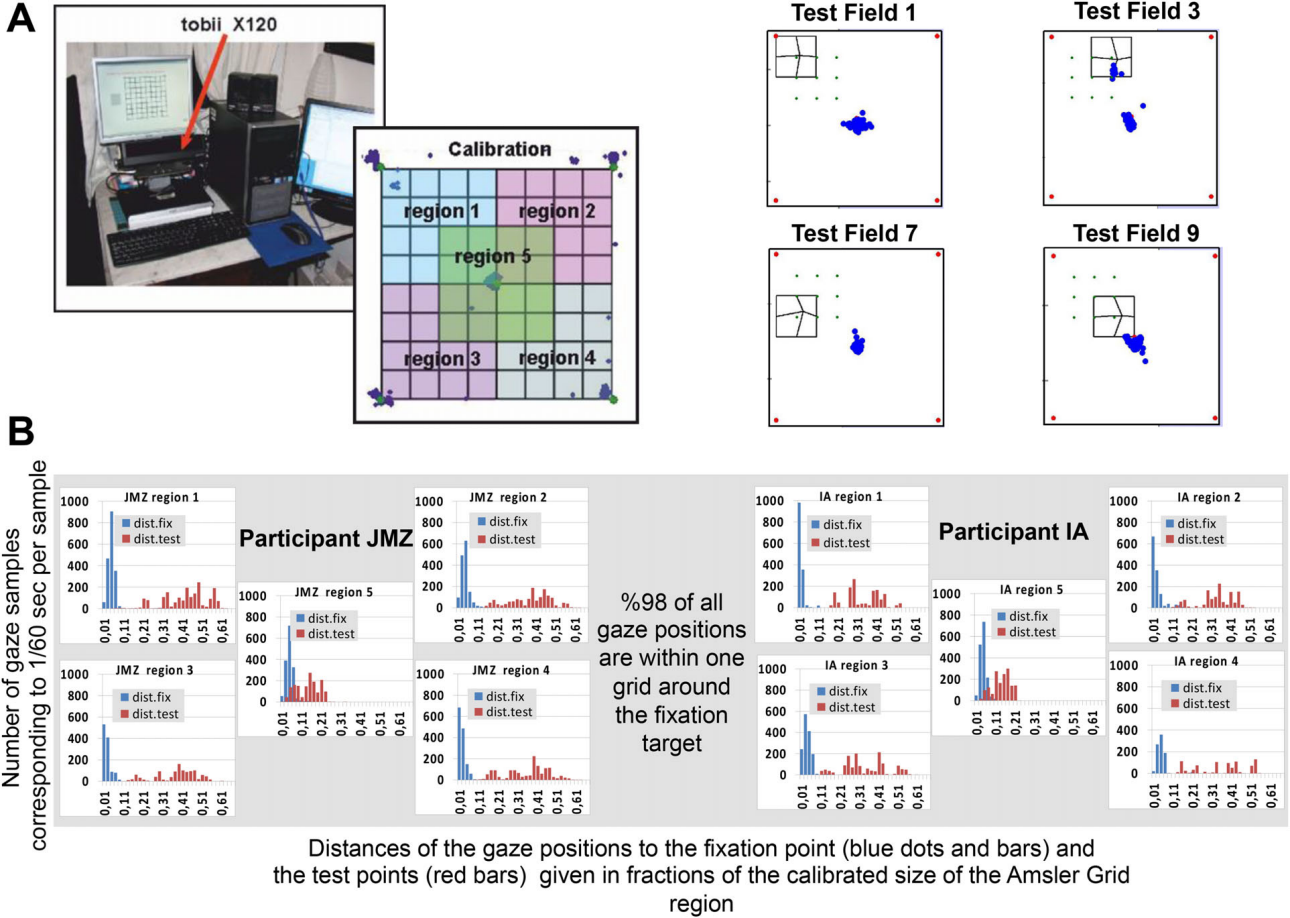
The main results on the gaze stability control during the An Iterative Amsler Grid (IAG) line alignment paradigm in experiment 1, where we tracked the eye fixations of two participants while performing the task. (A) Example data from one participant, which demonstrates the gaze positions to the fixation point (blue dots). Note that following a calibration phase on a regular Amsler Grid, gaze data were collected on all test points (Figure 1B) of different regions ((A) – on the left) during the IAG task, where participants fixated centrally. (B) Aggregated data from individual samples for four test regions and their nine test points. Results confirm that fixation could in fact be kept on the central point as indicated by the narrow distribution of the blue bars

Figure 3A shows histograms of the two distances for gaze positions on the calibrated stimulus region as seen by the participant when adjusting the grid for one eccentric test region and its four test fields, clearly indicating that ‐ apart from one short episode where the participant moves into test field 3 – steady fixation is maintained on the fixation target. Figure 3B shows the cumulative fixation distances for all nine fields and each of the five regions tested for both participants, confirming steady fixation of the fixation target: overall, 98% of all gaze positions are within the 2 by 2 squares of the Amsler Grid surrounding the fixation target. Note that in the aggregated data from individual samples, the distribution of the distances of gaze positions to the test points (red bars in Figure 3B) shows larger variations, as different test points were tested in each trial. What is critical here, on the other hand, is the distribution of the distances of gaze positions to the fixation point (blue bars in Figure 3B), which shows a narrow dispersion. This gives us confidence that fixation is maintained during the IAG procedure.

### 2.2 Experiment 2: IAG – a control position paradigm

In the first experiment, the task required participants to judge whether the vertical and horizontal line elements crossing the target point were straight or curved in chevron‐like stimuli. The sensitivity to this type of curvature detection is known to be in the hyperacuity range, where performance is sharper than the limits set by the spacing across adjacent cones.^27^, ^28^ High position acuity under certain conditions (i.e., in the vernier acuity judgments of spatial misalignment), however, has only been demonstrated in the presence of nearby reference stimuli, and thresholds have been shown to increase for configurations with large line separations.^27^

**FIGURE 4:**
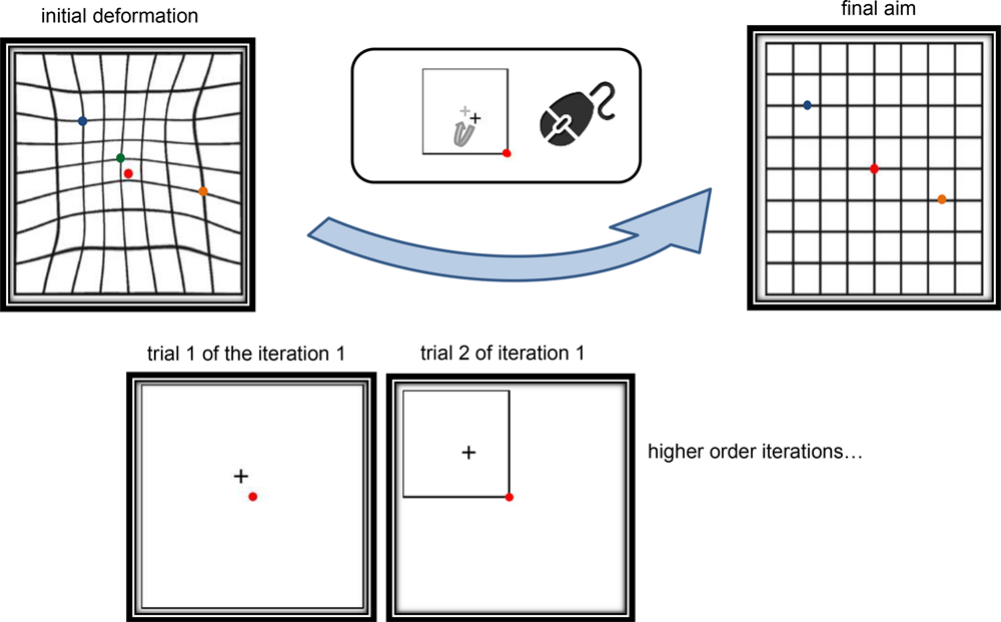
Visual depiction of the experiment 2, where on an interactive graphical user interface, participants were asked to drag a small cross point to the center of the reference square by using a mouse to correct for deviations from the regular Amsler Grid across iterations. Green, blue, and orange dots are the deviated test points at different spatial scales, which were to be corrected in the subsequent iterations. Red dot in the center is the fixation point. The sizes and eccentricities of the reference frames were the same as those in the experiment 1

Using both a three‐line bisection task, where participants judge the equidistance of a centralized target line with respect to the peripheral reference features and a vernier acuity task in the same setup, Klein and Levi^29^ demonstrated that the mechanisms underlying the bisection and vernier acuity tasks may be different. In fact, whereas displacement sensitivity and bisection acuity were shown to decline with age, vernier acuity was demonstrated to be independent of age, providing further support for the dissociated neural underpinnings.^30^, ^31^ Note that our IAG procedure in the first experiment is a curvature detection task. The second version that we developed here requires a two‐dimensional (vertical and horizontal) bisection judgment (Figure 4).

**FIGURE 5:**
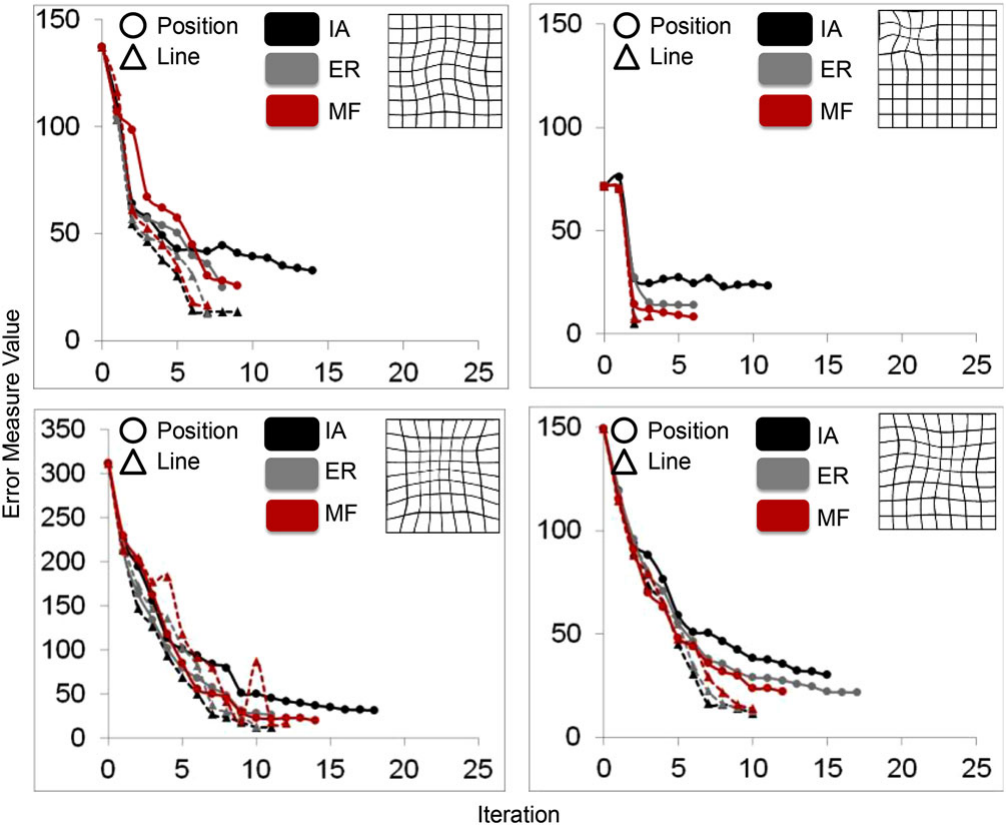
The main results of the position paradigm of experiment 2 (circles), where the error measure values are plotted as a function of iterations. Note that the results of experiment 1 from the same participants were also included in the graph for a comparison (triangles and dotted lines). Data from three participants are indicated as black, grey, and red circles and triangles. Whereas each graph shows the performance on a different template style presented on the right‐top (template 1, top‐left; template 7, top‐right; template 6, bottom‐left; template 2; bottom‐right), different colors are used to indicate different participants with initials specified in the legends. IA, ER, and MF stand for participant initials

Figure 5 shows the error measure values as a function of iterations for each participant tested on four deformation templates (templates 1, 7, 6, and 2) using the position paradigm (circles). The data from the same participants on the line alignment paradigm in experiment 1 (triangle) are also redrawn to make comparisons between the performances on two versions of the IAG paradigm. For all three participants, it can be seen on the graphs that the final error measure values are bigger when participants use the position paradigm (*M*_Template1 _= 28.00, *M*_Template7 _= 18.92, *M*_Template6 _= 21.81, *M*_Template2 _= 24.64; *M*_AllTemplates _= 23.34, standard error of the mean (SEM) = 1.94) than when they use the line alignment paradigm (*M*_Template1 _= 15.24, *M*_Template7 _= 8.74, *M*_Template6 _= 14.47, *M*_Template2 _= 14.60, *M*_AllTemplates _= 13.27, SEM = 1.52) to correct for the initial deformations. It can also be seen that it takes many more iterations to complete the task using the position paradigm (*M*_Template1 _= 10.33, *M*_Template7 _= 7.67, *M*_Template6 _= 14.33, *M*_Template2 _= 14.67; *M*_AllTemplates _= 11.75, SEM = 1.68) than the line alignment paradigm (*M*_Template1 _= 8.00, *M*_Template7 _= 2.67, *M*_Template6 _= 11.67, *M*_Template2 _= 10.33; *M*_AllTemplates _= 8.17, SEM = 1.98), which together suggest that participants took advantage of the alignment of the lines, as well as the position of the intersection points when they corrected for the deformations in experiment 1.

**FIGURE 6:**
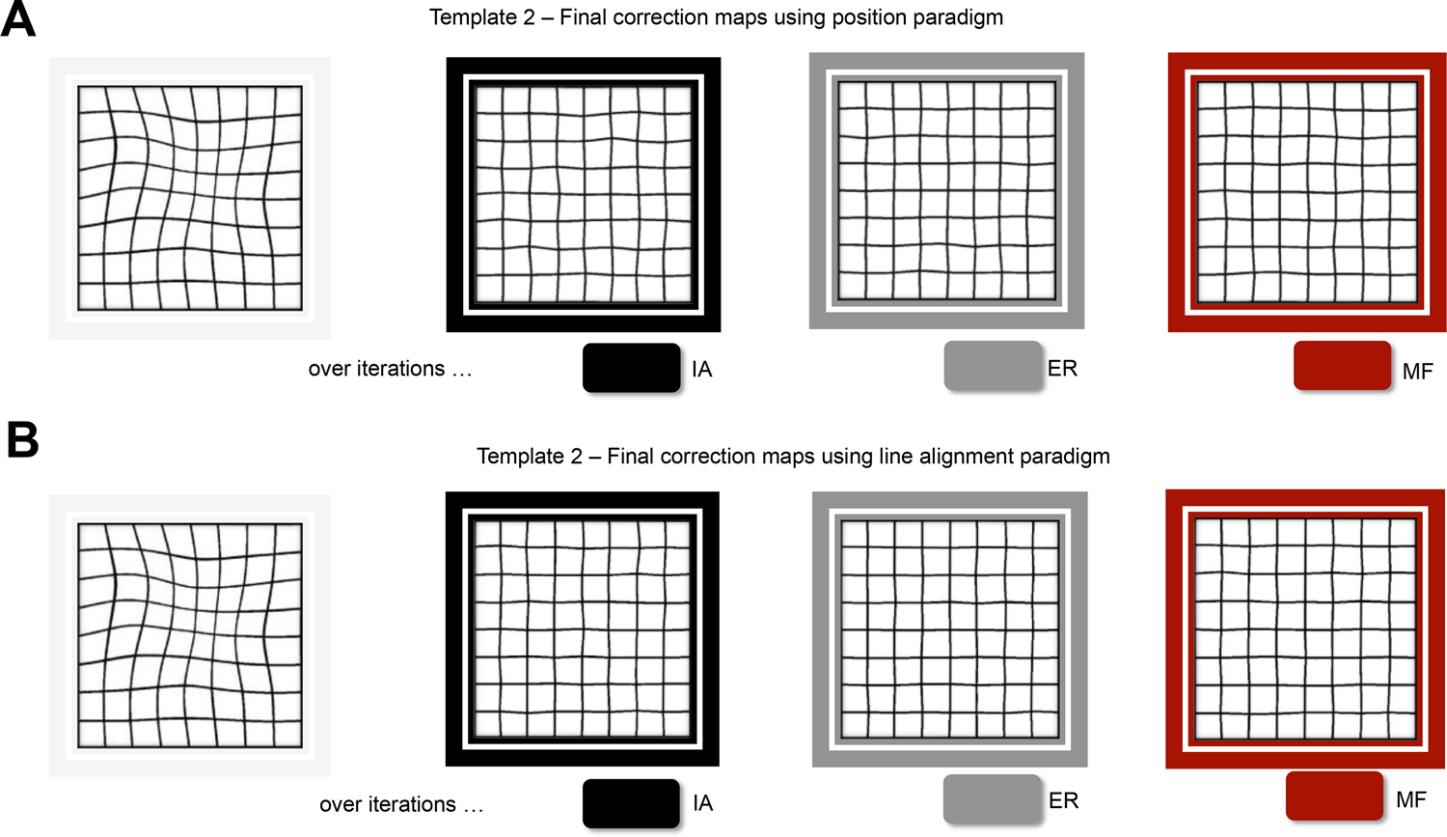
Final correction maps of template 2 in position (A) and line alignment (B) paradigms for each participant who participated in both tasks. Different colors are used to indicate different participants with initials specified in the legends. Although the end results seem to be very close to each other in position and line paradigms, it takes many more iterations to complete the task using position paradigm. IA, ER, and MF stand for participant initials

Figure 6 shows the final correction maps of Template 2 for all three participants using the position (6A) and line alignment paradigms (6B). A qualitative inspection over the images reveals that the spread of final errors does not depend upon the eccentricity and that the residual errors while positioning the node cross are not intensified over the regions further from the central fixation point. Thus, within the range of our test points (∼11.7 degrees), using 100% contrast stimuli (black cursor on a white background) and central fixation, although over many more iterations, in experiment 2 we can still obtain reliable maps using the position, as good as in the line alignment paradigm in experiment 1 and that the final correction maps end up to be very similar in both procedures.

## 3 GENERAL DISCUSSION

In this study, we tested a computer‐based “IAG” method that we developed to map deformations in different regions of the visual field. On a graphical user interface, where participants interacted with stimuli (either dots or lines in two different versions) using a mouse and a cursor, local regions of an Amsler Grid were tested at different spatial scales over multiple iterations. Our results indicate that:

1. Error measures values tend to approach zero over the higher iterations. Thus, healthy participants can successfully correct the simulated metamorphopsic deformations at different scales (local versus global) and eccentricities using the "IAG" procedure.
2. Eye tracking data provide evidence for the stability of the fixation, which indicates that healthy participants can also reliably maintain their eyes on the centralized fixation during the IAG procedure.
3. Local deformations take less iterations to correct than global deformations.
4. Plotting error measures as a function of iterations, the slopes in the line task are steeper than those in the position task, which indicates that judging whether two cross lines are straight or curved is an easier task than judging whether a dot is in the center of a square. As a result of this, the line task is completed in a lower number of iterations than the position task.
5. This novel objective method presented here was verified by testing healthy participants who generated quantitative measures of location and extend of image deformations as experienced in AMD patients. This could be a promising potential to develop clinical application for assessing the stage of AMD and planning interventions, which go beyond subjective patient reports.

In clinical settings, metamorphopsia has been traditionally diagnosed using printed Amsler Grids, where patients are asked to indicate whether straight lines appear curved or wavy. This procedure, though, can only make a qualitative categorization (i.e., presence or absence of the condition) and is far from providing a reliable map of deformations with a quantified measure of severity. Recently, Matsumoto et al.^20^ developed a method called M‐Charts, where patients are presented with lines composed of sequenced dots with varying spacing in‐between. Based on the observation that the deformed percept is more pronounced in lines with high‐density dots, researchers used the minimum angle of dot interval where patients stop perceiving the irregularities as an index of the degree of metamorphopsia. Although this method provides an objective index to quantify deformations; however, it does not deliver a topographical map of the perceived distortions. Here, we demonstrated that the IAG method based on human‐computer interaction can provide, for healthy participants and simulated image deformations, not only an index of severity, but also a reliable map of apparent irregularities.

Martin‐Gonzalez et al.^25^ used a similar image interpolation methodology as we applied here in a procedure where healthy participants described the simulated deformations verbally so that the examiner could correct them using an interpolation method on the digital image. In a more recent study, Wiecek et al.^26^ have also followed a similar procedure, where experimenter used the computer mouse to mimic deformations on a digitalized Amsler Grid according to the instructions given by the patients. In both of these studies, though, the authors used a complete Amsler Grid; thus, a deformed grid would always be observed in the presence of a contextual texture at a single spatial scale. The spatial scaling of the image, however, as well as the retinal eccentricity has been shown to be critical in determining the sensitivity to distortions.^6^ Conditions like epiretinal membrane are also known to affect processes at different spatial scaling to a varying extent, sensitivities being the worst for higher spatial frequencies.^32^ Here, our IAG procedure scans regions of the visual field using grids with different spatial scales, allowing corrections at coarse, as well as fine scaling, which seems to be crucial for diagnostic purposes.

It is known that both the separation and the eccentricity of the stimulus features may be important in determining the precision of spatial localization.^29^ Klein and Levi demonstrated that for large stimulus separations or eccentricities, as was used in our experiments here, the position thresholds vary in agreement with the cortical magnification factor. In fact, when scaled according to the cortical magnification factor, vernier acuity was demonstrated to be as good in periphery as it is foveally.^33^ The results about the effect of eccentricity on the thresholds are inconsistent, though, as some studies have demonstrated that with large and fixed separations when thresholds are plotted as a function of eccentricity, there is merely a change in thresholds with eccentricity.^34^, ^35^ In fact, irrespective of the type of the task (position or line alignment paradigm), here, our results demonstrated that participants could make corrections in the peripheral, as well as in the central grids, although peripheral points could require further iterations for finer corrections. This demonstrates that our method is not limited by vernier thresholds, that is, correcting deformation is sufficient in the supra‐vernier domain. Moreover, wet AMD patients with metamorphopsia were shown to have fewer distortions on the periphery than in the center,^19^ which implies that testing eccentric regions is a rare need in the clinic.

In our IAG setup, participants found the position paradigm more difficult than the line alignment paradigm as indicated by the bigger final error values and iterations for the former. It is important to note that our line alignment paradigm was a curvature detection task, where horizontal and vertical lines dissected by the target cursor acted as contours, providing spatial reference frames for the position percept. In a study which aimed at investigating the role of primary visual cortex in visuospatial integration, Kapadia et al.^36^ have shown that the contextual flanking stimuli, aligned with a central target orientation significantly increase the response rates to the central stimulus in comparison to the baseline levels measured in the absence of collinear flanks. This collinear facilitation is regarded as an important element of contour perception^37^ and has been suggested to be mediated by the long‐range connections in the primary visual cortex,^38^, ^39^ where extra‐receptive‐field stimuli modulate the responses to those in the classical receptive fields (for a more detailed discussion, see Loffler^40^). Here, distinctively from the line alignment paradigm, in the position paradigm of IAG, on the other hand, participants were presented with a target cursor in isolation. Thus, in this setup, the task was rather like a bisection task, with no facilitation effects involved, which increased the thresholds and error measures. In the light of these results, it would be better to test patients on the line alignment paradigm than the position paradigm, if more reliable maps are aimed to be obtained. These results are in line with the findings by Wiecek et al.,^26^ where they also used a shape discrimination and vernier hyperacuity task, in which AMD patients were asked to adjust the corner points of a notional square until it appeared straight, as well as a digitalized Amsler Grid task with contextual lines. Similar to our current results, they demonstrated that the accuracy of the grid task at distinguishing between patients and control participants was higher than that of the hyperacuity task, where reference grids are lacking.

Our computer‐based IAG method provides a means of obtaining a map of perceptual deformations in the presence of a central fixation. The quantification of metamorphopsia is a problem extensively studied in the literature not only in AMD but also in epiretinal membrane, reattached retinal detachment, and macular hole.^20^, ^41^, ^42^ Arndt et al.^41^ have demonstrated that the decrease in the area of visual distortion measured by an Amsler Grid exploration strategy based on scanning laser opthalmoscope was correlated with the change in visual acuity after the removal of the epiretinal membrane with surgery. Using a new computerised method to measure metamorphopsia called MonPack One, where a local 4 × 4 grid pattern of straight or curved lines are presented at different regions of the visual field across trials for the patient to report whether the grid is normal or distorted, Lee et al.^43^ also reported that the metamorphopsia scores obtained by the MonPack One method were significantly correlated with the retinal thickness and the N1 and P1 amplitudes of multifocal ERG (electroretinogram), particularly in the central subfields of the patients with epiretinal membrane. These results were contradicted by Matsumoto et al.,^20^ who demonstrated that using a methodology called M‐Charts, there was not a relationship between the metamorphopsia score and the visual acuity of ERM patients. The authors argued that the quantification of metamorphopsia may provide new clinical information irrespective of the visual acuity. In fact, Wittich et al.^42^ have also provided no evidence for a correlation between the magnitude of visual deformations and visual acuity following the surgery. Although the exact relationship between the amount of metamorphopsia and the anatomic and functional outcome of these retinal diseases is yet to be discovered in the clinical settings, obtaining an accurate map of the deformations may allow us, in the future, to develop a computerised aid, where an eye tracker simultaneously keeps track of the eye fixation position, and digital images are warped instantaneously according to the patients’ deformation maps to correct for perceptual distortions (i.e., while reading). To this end, it is important to ensure a stabilized fixation during the IAG procedure. Here, we demonstrated that healthy participants could maintain fixations reliably during the IAG task. Thus, deformation maps obtained from this procedure may be potentially used as an input to a computerized reading aid. It is important to note, however, that the control data which we collected here were under binocular viewing conditions and that the patient testing would have to be carried out monocularly for each eye which typically would be affected by different image deformations, separately, and implemented in a stereoscopic setup to build such technologies in the future.

Obtaining a reliable map of deformations having a potential in opening new horizons in developing technologies that would potentially increase the quality of life of patients with macular diseases, it is not surprising that there have been an increasing number of reports in computer‐based metamorphopsia measurement methodologies in the literature in the past few years. One such method called the line sag test (LST) is a computerized psychophysical test in which the observer is presented briefly with a curved line and asked to report whether the line appears to bulge inwards or outwards.^44^ Using a staircase procedure, an algorithm neutralizes the amount of perceived sag by presenting a curve at the opposite direction to the response of the patient in the next trial. Comparing the results to those obtained with a traditional Amsler Grid, the authors concluded that the LST could capture more cases of deformations in patients with ERM. In another method, called MacuFix, patients are presented with four square fields of 4 × 4 grids and are required to report the one with the most distorted pattern.^45^ An algorithm determines the smallest detectable magnitude difference according to the answers across trials. It has been demonstrated that MacuFix reliably produced higher values of metamorphopsia scores in patients with macular edema than those with normal macula.^46^ In both of these methodologies, however, patients were tested for deformations at a single scale with a fixed stimulus size. The procedure we introduce here has the potential to test not only the effect of background grid (either present of absent), but also the deformations at different scales.

Preliminary data from patients at various stages of AMD demonstrated that whereas extensive scotomas or serious fixation problems are a challenge for the use of IAG procedure, patients in the early stages of their diagnosis can generate reliable deformation maps that correspond to their subjective reports. In fact, Martin‐Gonzalez et al.^25^ had also used a similar procedure where the healthy examinee described the simulated deformations verbally, which was then corrected by the researcher using a digital image interpolation technique. Similarly, Wiecek et al.^19^ also introduced a computer‐based Amsler Grid task, where the description of the extent and shape of distortion by the participants with maculopathy was used to correct for the deformations by the researcher using a computer mouse. In our case, even for those patients, who were not confident using the computer mouse due to motor control problem or lack of practice with the computers, it was still possible to generate maps in a setup, where the experimenter adjusted the tilt of line segments according to the oral description of patients. In the initial phases of the disease, as the small scotomas might be filled‐in or perceptually completed,^47^ the IAG task has still proved itself to be a reliable measure to map deformations. In some cases, however, AMD coexisted with other eye conditions which affected the visibility or the stabilization of fixation, making it more difficult to report the tilts. Thus, a variety of factors, including scotomas, and other eye conditions are to be considered while testing patients in the future.

All in all, the graphical user interface IAG, which we developed, here, could hold promise to allow the quantification and mapping of visual deformations in clinical conditions such as macular degeneration and epiretinal membrane. Future clinical studies will be needed for a more systematical investigation using a larger and diverse patient sample to improve and apply the procedure for diagnostic purposes.

## 4 METHODS

### 4.1 Experiment 1: IAG ‐ *line alignment paradigm*

#### 4.1.1 Participants

Nine adults participated (five females, four males), eight of which were naïve to the purpose of the experiment. Participants were undergraduate and graduate students, postdoctoral researchers, and instructors at Royal Holloway, University of London. Visual acuity was normal or corrected‐to‐normal for all observers. The mean age of the participants was 32.11 years old with a range of 28–45. Before starting the testing, all participants signed an informed consent form which provided detailed information on the nature of the study. The experiment was conducted in accordance with the ethical guidelines laid down by the RHUL Department of Psychology Ethics Committee and adhered to the tenets of the Declaration of Helsinki.

#### 4.1.2 Apparatus

Observers were seated 50 cm from a Compaq Presario CQ57 Laptop monitor, with a refresh rate of 60 Hz. Stimuli were generated using the main functions in Matlab R2012a. The dimensions of the screen were 34.5 cm × 19.5 cm. At this distance, the screen subtended 38 × 22 degrees. The laptop was elevated so that the fixation spot appeared at eye level. The responses were collected using a laser mouse.

#### 4.1.3 Stimuli

Stimuli were based on 8 × 8 grid of square horizontally and vertically oriented black lines (with 1 mm width) on a white background, of the notional Amsler Grid with dimensions of 10.5 cm × 10.5 cm, corresponding to 12 × 12 degrees at a viewing distance of 50 cm, with a red fixation target (with a diameter of 0.5 cm) always being visible at the center. In this frame, we presented as square test field with a given test point at the position of the deformed grid (green, blue, and orange in Figure 1A) which was connected by lines to the four corners of the test field. The size of the test fields could be the size of the full notional Amsler Grid (8 × 8 squares, first trial of iteration 1), half of it (4 × 4 squares, 6.1 × 6.1 degrees, second trial of first iteration), or a quarter of it (2 × 2 squares, 3.1 × 3.1 degrees, all higher order iterations). In each iteration, 3 × 3 adjacent test points were presented at the appropriate scale (see Figure 1B). The largest grid with dimensions of 10.5 cm × 10.5 cm (corresponding to the overall size of the notional Amsler Grid) and the medium‐sized grids with dimensions of 5.3 cm × 5.3 cm were used in the first and second iteration of the IAG procedure, respectively, and the small‐sized grids with dimensions of 2.7 cm × 2.7 cm were used in all subsequent iterations.

Participants were tested on 10 different 8 × 8 square template grids which all held a deformation field simulating a patient's sight suffering from macular degeneration. Deformation fields on those template grids were retrieved from images in scientific journals, as well as from the database of Dr Edward Doyle. Whereas six of the 10 template grids had a deformation field spread all over the visual field; four of them had a deformation field at a single region (top‐left, top‐right, bottom‐left, and bottom‐right) to control for anisotropic effects.

#### 4.1.4 Procedure

In experiment 1 (see Figure 1A), the task of the participants was to fixate in the middle and adjust an 8 × 8 deformed template grid so as to appear straight to them. In nine trials of the initial iteration, a node point of a 2 × 2 grid was presented sequentially on the positions of the corresponding template points (Figure 1B). During the interactive phase of the experiment, under binocular viewing, participants used a mouse to change the positions of the nodes such that the horizontal and vertical lines intersecting at the node point were perceived to be a perfect cross (+). In two different versions of the program, the mouse cursor was either visible or invisible on the screen. The mouse invisible condition was introduced to control for the eye movements and attentional shifts from the central fixation point to the mouse cursor. To change the position of a node, participants had to move the mouse while keeping its left button pressed. Once they were satisfied with the outcome, the next grid was presented on the release of the button. To avoid involuntarily mistakes in the initial iteration, if a participant released the mouse button when the node had been at a point outside the borders of the 2 × 2 grid, the same node was tested again in the next trial. The data points were then fed into a biharmonic spline interpolation method implemented in Matlab 4 grid data function (with an option "v4," supporting two‐dimensional interpolation) to produce an updated image of the template grid with corrected values sitting on the test points. This biharmonic spline interpolation method is based on an algorithm, where the aim is to find the minimum curvature surface that passes through a set of non‐uniformly spaced data points, in this case the intersection points corrected on the Amsler Grids.^44^ In the higher order iterations, participants could continue with further and finer corrections by selecting a certain region on the updated image, which was presented at the end of each iteration. The positions of the intersection nodes on the interpolated grids were then fed into the incoming trial as the initial test point positions. There were nine regions to select from: top‐left, top‐central, top‐right, middle‐left, middle‐central, middle‐right, bottom‐left, bottom‐central and bottom‐right. Each of these regions was consisted of nine test points, which together covered all the points in the template grid. The border points were assumed to be fixed at their straight positions. Should a participant make a mistake at any point, they could select the region that covers that point‐to‐be‐corrected in the next iteration. Observers continued the task until they were satisfied that the updated image was a good approximation to a non‐deformed Amsler Grid (for the code flow chart, refer to chart S2).

Experiment 1 had a between‐subjects design, where different participants were tested on the 8 × 8 template grids in either mouse visible or mouse invisible conditions, except for one participant (IA) who was tested on both. Whereas in the mouse visible condition, the mouse cursor was visible on the screen, in the mouse invisible condition it was absent from sight, the comparison of which could give us an insight about the role of attention. The presentation order of the templates was semi‐randomized such that each half of the experiment consisted of three wide‐spread and two region‐specific deformations.

### 4.2 Experiment 2: IAG – a control position paradigm

#### 4.2.1 Participants

Three adults (one female, two males) who participated in experiment 1 also participated in the control experiment: experiment 2. The mean age of the participants was 28.66 years old with a range of 25–32. Two of the participants were naïve to the purpose of the experiment. Visual acuity was normal or corrected‐to‐normal for all observers. The experiment was conducted in accordance with the ethical guidelines laid down by the RHUL Department of Psychology Ethics Committee.

#### 4.2.2 Apparatus

Experiment 2 was carried out on the same apparatus as the experiment 1 on a Compaq Presario CQ57 Laptop monitor, with a refresh rate of 60 Hz. Stimuli were generated using the main functions in Matlab R2012a.

#### 4.2.3 Stimuli

To test whether participants make corrections based on the alignments of the horizontal and vertical lines or the positions of the node points sitting on the intersection, in experiment 2, we used a cursor stimulus which was a black cross (with 7 mm width and height) inside a square frame. The dimensions of the stimuli were as same as the dimensions of the stimuli in experiment 1.

#### 4.2.4 Procedure

The procedure in experiment 2 was similar to that of experiment 1, except that participants used the mouse to drag the cursor stimulus such that it sat in the center of the test square (Figure 4). Thus, whereas experiment 1 was a line alignment paradigm, and experiment 2 was a dot‐positioning paradigm. The dimensions and the eccentricity of the squares changed across different blocks of iterations with the same as in experiment 1. Participants were tested on template 1, template 2, template 6 and template 7 of experiment 1. The presentation order of the templates was randomized.

## 5 CONCLUSIONS

Metamorphopsia, a condition experienced in AMD, is characterized by perceived distortion of a shape. In metamorphopsia, straight lines appear to be curved and wavy to patients with AMD. Here, we tested the state‐of‐the‐art of a computer‐based “IAG” method that we developed to map visual deformations in different regions of the visual field. In healthy participants, we verified it to generate quantitative measures of image deformations as experienced in AMD patients. This has the potential to inform future clinical application to assess AMD and guiding interventions, going beyond subjective patient reports.

## CONFLICTS OF INTEREST

The authors declare no conflicts of interest.

## ETHICS APPROVAL

The experiment was conducted in accordance with the ethical guidelines laid down by the RHUL Department of Psychology Ethics Committee and adhered to the tenets of the Declaration of Helsinki. All volunteered participants signed an informed consent before the experiment, which clearly described the experiment and their rights.

## AUTHOR CONTRIBUTIONS

Conceptualization of the project and methodology: Johannes Zanker. Coding: Inci Ayhan. Data collection: Inci Ayhan and Johannes Zanker. Patient data and improvement of the methodology: Edward Doyle. Original draft writing and editing: Inci Ayhan and Johannes Zanker. Data analyses: Inci Ayhan and Johannes Zanker. Proofreading: Johannes Zanker, Edward Doyle, Inci Ayhan.

## Supporting information

Supplementary information

Supplementary information

## Data Availability

All data are available from the corresponding author upon request.
