# Supplementary material for: Measuring image distortions arising from age‐related macular degeneration: An Iterative Amsler Grid (IAG)
Source: MedComm (2020). 2022 Mar 4;3(1):e107. doi: 10.1002/mco2.107 (PMC8906453; doi:10.1002/mco2.107)
Supplement: Supplementary file 1 — Supplementary information [file MCO2-3-e107-s002.docx]

**Measuring image distortions arising from age-related macular degeneration:**

**An Iterative Amsler Grid (IAG)**

**Authors**

Inci Ayhan^1^, Edward Doyle^2^ & Johannes Zanker^3^

1 Department of Psychology

Boğaziçi University, Istanbul, Turkey

2 Department of Ophthalmology

Torbay Hospital, Torquay, UK

3 Department of Psychology

Royal Holloway University of London, Egham, UK

Corresponding Author: Johannes Zanker, [j.zanker@rhul.ac.uk](mailto:j.zanker@rhul.ac.uk)

Department of Psychology, Royal Holloway University of London

**Supplementary Material**

**
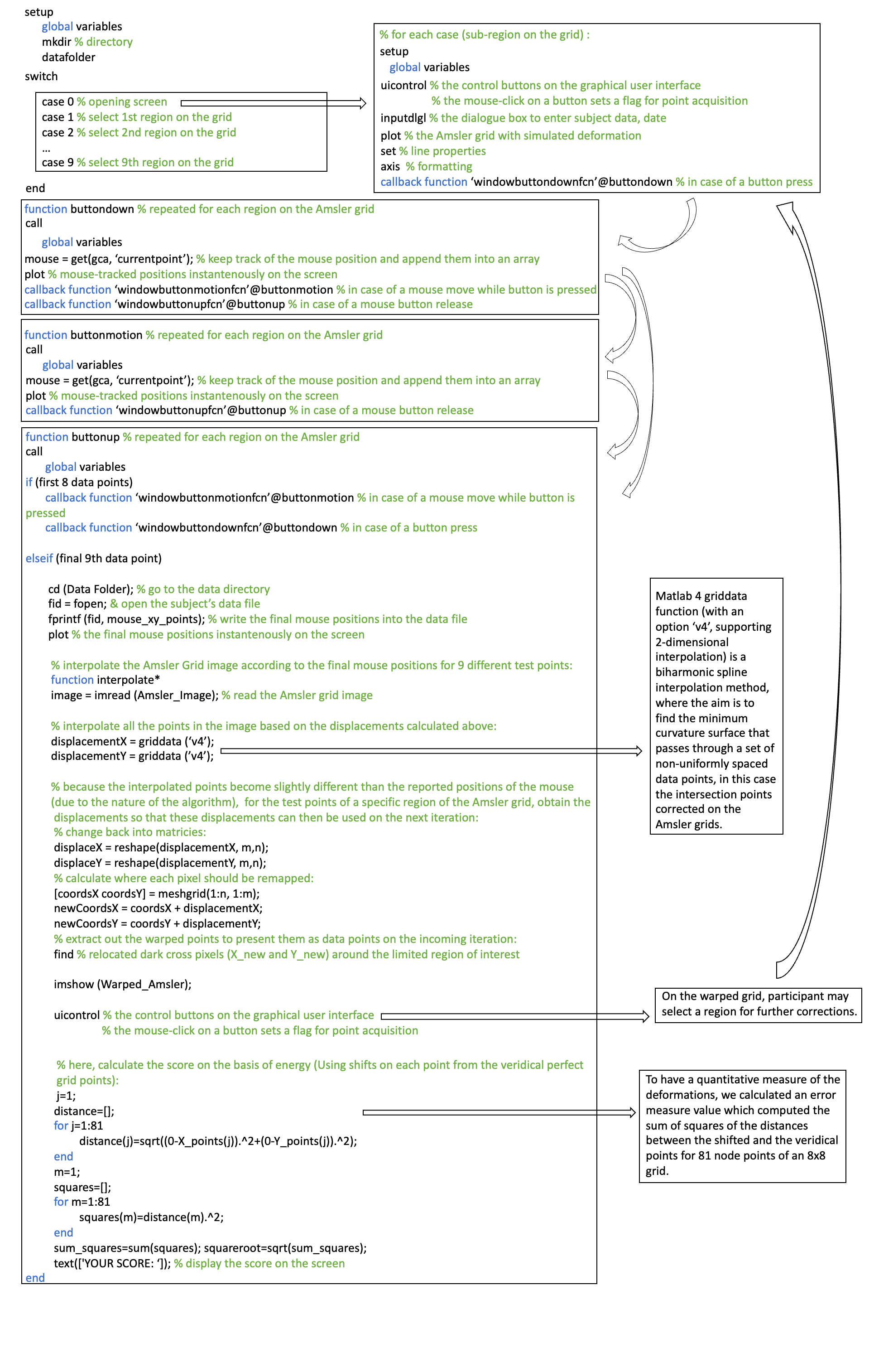
**

*Reference (for the image warping function): function warpImage.m by the GitHub User Standford Center for Earth Resources Forecasting (signed off by Jef Caers) https://github.com/SCRFpublic/TSSIM/blob/master/tssim/warpImage.m
